# Acute respiratory distress syndrome secondary to COVID-19 mRNA vaccine administration in a pregnant woman: A case report

**DOI:** 10.5339/qmj.2022.40

**Published:** 2022-08-09

**Authors:** Betsy Abraham, Hassan Mohammed Saeed, S. A. Azeez Pasha

**Affiliations:** ^1^Department of Intensive Care, Salmaniya Medical Complex, Bahrain E-mail: dr.betsy.a@gmail.com

**Keywords:** Acute respiratory distress syndrome, ARDS, Vaccine adverse event, mRNA vaccine, Vaccine-associated adverse event, COVID-19

## Abstract

Healthcare professionals monitoring vaccine-related adverse responses should be aware of COVID-19 vaccine-related acute respiratory distress syndrome to enable expeditious diagnosis and treatment. We report the first case of acute respiratory distress syndrome in a young pregnant woman, occurring immediately after a second dose of the Pfizer-BioNTech BNT16B2b2 mRNA COVID-19 vaccine and requiring a brief period of mechanical ventilation, with a good response to a two-week steroid course. She had recovered from mild COVID-19 infection requiring home isolation nine months prior to the current vaccination dose. Without depreciating the colossal benefit of the COVID-19 vaccine, vaccine-related acute respiratory distress syndrome should be listed as a possible adverse reaction.

## Introduction

Sporadic vaccine-related adverse events are pertinent to the treating physician but do not depreciate the colossal benefit of vaccination and the well-validated safety profile of the Pfizer-BioNTech BNT16B2b2 mRNA vaccine.^1,2^

Paroxysmal ventricular arrhythmia, right leg paresthesia, shoulder injury related to vaccine administration, right axillary lymphadenopathy, and interstitial pneumonitis are the reported serious adverse effects associated with this vaccine.^3^ Nevertheless, post-authorization safety study reports of severe reactions have been exceptionally sparse, occurring in only 4.5 reported cases per million doses.^4^ It is important to evaluate the safety of COVID-19 vaccines, especially in the pregnant population, as clinical trials only enroll healthy, young, nonpregnant individuals. We present the first case report of acute respiratory distress syndrome in a young pregnant woman after a second dose of the Pfizer-BioNTech BNT16B2b2 mRNA COVID-19 vaccine. The patient's consent was obtained for the publication of this case report, and there is a waiver of Institutional Board Research Review Approval for case reports at our institute.

## Case Report

**Figure 1.**
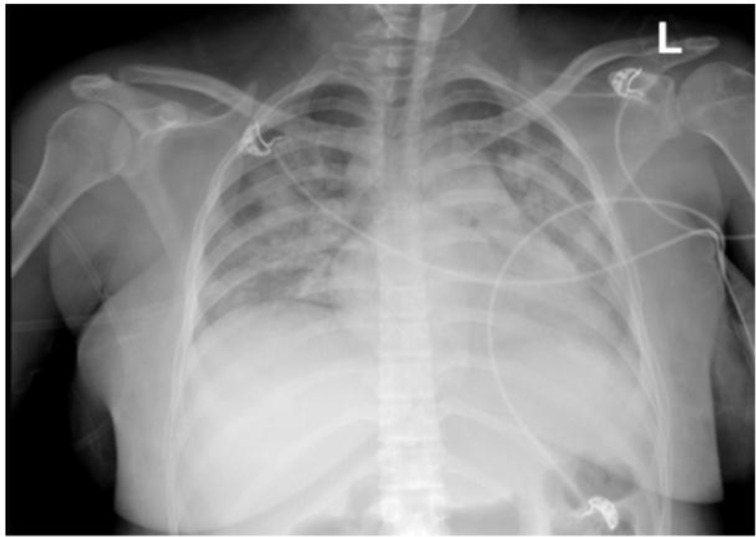
Chest X-ray revealing right lower lobe consolidation with extensive diffuse infiltrates.

A 24-year-old female with Gravida 4 Para 3 pregnancy at 25 ± 2 weeks presented with a 5-day history of fever, bilateral chest pain pleuritic in nature, exertional shortness of breath, sweating, and palpitation starting 1 day after she received her second dose of the Pfizer-BioNTech COVID-19 vaccine. She had recovered from a mild COVID-19 infection (RT-PCR cycle threshold value: 25.7) requiring home isolation nine months prior to the current vaccination dose and was still on thyroxin 100 micrograms for hypothyroidism. In the emergency department, she was afebrile and hypotensive, with 80%–85% desaturating oxygen saturation (SPO2), bilateral bronchial breath sounds, and crepitations. A chest X-ray (Figure–1) revealed right lower lobe consolidation with extensive diffuse infiltrates. A computed tomography pulmonary angiogram scan (2) showed a dilated pulmonary trunk (3.35 cm), right lower lobe consolidation with an associated bilateral diffuse area of ground glass consolidation, a few enlarged mediastinal lymph nodes, and mild pleural effusion, with no major pulmonary embolism. Her white blood cell count was 22.09 ×  10^9^/L, with neutrophils 77%, lymphocytes 10%, monocytes 4%, band forms 8.0%, atypical lymphocytes 1.0%, and a C-reactive protein level of 344.66 mg/L. Her thyroid stimulating hormone, electrolytes, platelet count, coagulation profile, and liver and renal function tests were within normal limits.

On day 2 of admission to the intensive care unit, the echocardiogram showed normal left ventricular systolic function, an ejection fraction >55%, and normal valves with no evidence of raised pulmonary artery pressure or left ventricular clot or pericardial effusion. The cardiology referral team recommended initiation of a therapeutic dose of enoxaparin 80 mg twice a day for myocarditis in view of nonspecific ST-T changes and a mild rise in troponin-I (4.700 ng/mL). She was electively intubated on day 2 because of persistent tachypnea and a PaO_2_/FiO_2_ ratio of ≤ 100 (PEEP ≥ 5 cmH_2_0) on a high-flow nasal cannula and noninvasive ventilation. Antibiotics were escalated to meropenem 1 g thrice daily and vancomycin 1 g twice daily in view of worsening desaturation, a white blood cell count of 22.09 ×  10^9^/L and C-reactive protein 356 mg/L. She was also started on prednisolone 1 mg/kg/day once daily. Three serial endotracheal tube secretion cultures (days 1, 3, and 5), two serial blood cultures (days 1 and 4) and a urine culture (day 1) were sterile. RT-PCR for *Mycobacterium tuberculosis*, an acid-fast bacilli smear, and pneumocystis jirovecii antigen from deep tracheal aspirate were negative. Four serial samples analyzed by COVID-19 RT-PCR (days 1, 2, 3, 5, and 7) and two COVID-19 antigen tests (days 1 and 2) were negative. She was seronegative for *Legionella pneumophilia*, *Mycoplasma Pneumoniae*, *Coxiella Burnettii*, *Chlamydia Pneumoniae*, Adenovirus, respiratory syncytial virus, Influenza A, Influenza B, Parainfluenza IgG, and IgM antibodies. A throat swab culture was negative for GROUP A, C, and G *Streptococcus*. A high vaginal swab was negative for *Trichomonas vaginalis*, *Gardnerella Vaginalis*, *Candida* Spp., and Group B *Streptococcus*. Serological tests for cytomegalovirus, Epstein-Barr virus, hepatitis B, hepatitis C, and human immunodeficiency virus were all negative. Screening tests for anti-nuclear antibody, complement, anti-cardiolipin, anti ß2-glycoprotein, anti-phospholipid antibody, and rheumatoid factor were all negative. Consecutive procalcitonin levels sent for 10 days (days 1–10) were negative. She was extubated on day 8. The patient's relatives refused consent for lung biopsy and bronchoalveolar lavage, which may aid in the diagnosis of drug-induced interstitial lung disease/hypersensitive pneumonitis, in view of her improving parameters. Prednisolone was tapered and stopped over two weeks from the day of initiation, with no relapse. She was discharged to the ward on day 11 on oxygen-nasal cannula 3L/min and discharged from the hospital on day 14 on room air with good fetal movements. She eventually gave birth to a healthy male baby through a normal vaginal delivery at term.

## Discussion

According to current Centre for Disease Control guidelines, pregnant women with a recent COVID-19 infection may consider delaying their first or second COVID-19 mRNA vaccine booster dose by 3 months from symptom onset or a positive test.^5^ A two-dose regimen of the Pfizer-BioNTech BNT16B2b2 mRNA COVID-19 vaccine separated by a 3–8-week interval provides 95% efficacy at preventing serious illness in expectant mothers.^6^ The most frequently reported adverse effects of the Pfizer-BioNTech BNT16B2b2 mRNA COVID-19 vaccine among pregnant women are fatigue, migraines, shivers, malaise, rash, and vomiting, the incidence of which increases after the second dose. Up to 89% of expectant mothers have reported injection site discomfort after the second dose of the vaccine.^7^

The occurrence of acute respiratory distress syndrome is linked with various infectious diseases, and there are no definitive reports implicating a temporal correlation of acute respiratory distress syndrome after vaccination with currently licensed vaccines.^8^ Pharmacovigilance systems such as the Vaccine Adverse Event Reporting System, the V–safe COVID-19 Vaccine Pregnancy Registry, Vaccine Safety Datalink, the Clinical Immunization Safety Assessment Project, and the Birth Defects Study to Evaluate Pregnancy Exposures (BD-STEPS) are actively monitoring COVID-19 vaccine safety during pregnancy.^5^ The World Health Organization (WHO) global pharmacovigilance database VigiAccess^TM^, as of 15 November, 2021, reveals 431 suspected COVID-19 vaccine-related acute respiratory distress syndrome cases. The major constraints of this database are the varied sources of information, the nonavailability of vaccine-specific data and unsettled causal association between a suspected adverse effect and the drug, differences in international coding criteria, errors, and a lack of transparent diagnostic criteria.^9^ Analysis of safety surveillance data on 10,162,227 mRNA vaccinated individuals from Vaccine Safety Datalink showed 12 cases of acute respiratory distress syndrome.^10^

Documented allergic responses to vaccines are not currently ascribed to the active ingredients but to inactive excipients that are added to enhance the vaccine potency, prevent bacterial contamination, and galvanize the immune response. The Pfizer-BioNTech mRNA COVID-19 vaccine is formulated with excipient polyethylene glycol to stabilize the lipid nanoparticles containing the mRNA, which itself has never before been utilized.^11^ Systemic reactions are more frequently noted in young individuals than in older ones and after the second dose than after the first dose of an mRNA COVID-19 vaccine.^11,12^ There is no evidence of a plausible correlation between vaccine-associated enhanced disease and authorized COVID-19 vaccines in controlled trials.^13^ Anaphylaxis has been noted more often immediately post administration of mRNA COVID-19 vaccines than with influenza vaccines and some others.^10^

Drug-induced interstitial lung disease is a diagnosis of exclusion and has a wide spectrum of clinical manifestations, from transient lung infiltration to acute respiratory distress syndrome. The *identification* and *singularity* criteria propounded by Camus et al.^14^ were fulfilled in this patient, as the COVID-19 mRNA vaccine was the only drug dispensed prior to symptom onset.^12^ The *temporal eligibility* criteria were fulfilled, as the patient developed respiratory symptoms or fever following vaccination. In this patient, we could not justify the rechallenge test, which we strongly discourage. The radiological picture and microbiological and serological test results ruled out connective tissue disorders, fulfilling the criterion of *exclusion of other conditions.* Due to nonavailability of patient consent and inconsistent findings, we did not execute bronchoalveolar lavage and lung biopsy. This case met the category of Level 1 confirmed evidence of the Brighton Collaboration Case Deﬁnition of acute respiratory distress syndrome as an adverse event following immunization.^8^ This case also met the WHO-causality assessment – B1(indeterminant-consistent temporal relationship but insufficient definitive evidence that the vaccine caused it/may be related to a new vaccine linked event) criterion.^15^

The clinical features of influenza vaccine-related lung diseases are similar to those of this case and include a short duration of presentation after vaccination, fever, bilateral ground glass opacities on chest CT with a bilateral nonsegmental distribution, and a good response to steroid therapy.^12^ This patient shared similar features with the eight patients who developed lung injury post COVID-19 vaccination and responded favorably to steroids.^3,12,16-18^ A past SARS-CoV-2 infection may have evoked a vigorous immune response after vaccination, resulting in respiratory distress syndrome in our patient. Hence, it is also plausible that this patient's diagnosis was purely fortuitous. Nevertheless, this report is vital both for pharmacovigilance and as a basis for future patient assessment.

## Conclusion

Healthcare professionals monitoring vaccine-related adverse responses should be aware of COVID-19 vaccine-related acute respiratory distress syndrome to enable expeditious diagnosis and treatment. Acute respiratory distress syndrome in a young pregnant woman, possibly secondary to a second dose of the Pfizer-BioNTech BNT16B2b2 mRNA COVID-19 vaccine and requiring a brief period of mechanical ventilation, responded well to steroids. Without depreciating the colossal benefit of COVID-19 vaccine, vaccine-related acute respiratory distress syndrome should be listed as a possible adverse reaction.

## Figures and Tables

**Figure 2.**
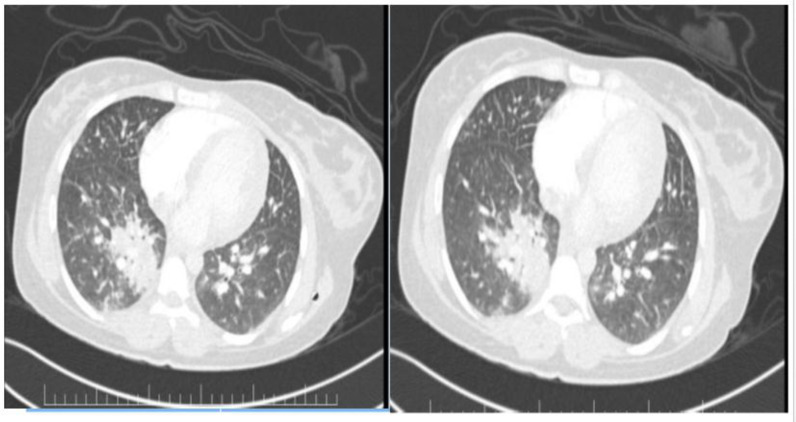
Computed tomography pulmonary angiogram scan – dilated pulmonary trunk, right lower lobe consolidation + associated bilateral diffuse area of ground glass consolidation + few subcentimeter mediastinal lymph nodes and mild pleural effusion + no major pulmonary embolism.

## References

[5] https://www.cdc.gov/vaccines/covid-19/downloads/covid-19-immunization-schedule-ages-5yrs-older.

[9] http://vigiaccess.org.

